# Differential Effects on the Translation of Immune-Related Alternatively Polyadenylated mRNAs in Melanoma and T Cells by eIF4A Inhibition

**DOI:** 10.3390/cancers14051177

**Published:** 2022-02-24

**Authors:** Biswendu Biswas, Ramdane Guemiri, Mandy Cadix, Céline M. Labbé, Alina Chakraborty, Martin Dutertre, Caroline Robert, Stéphan Vagner

**Affiliations:** 1 Institut Curie, PSL Research University, CNRS UMR 3348, INSERM U1278, 91401 Orsay, France; biswendu.gustaveroussy@gmail.com (B.B.); mandycadix@hotmail.com (M.C.); celine.labbe@curie.fr (C.M.L.); alina.chakraborty@curie.fr (A.C.); martin.dutertre@curie.fr (M.D.); 2 Biologie de l’ARN, Signalisation et Cancer, Université Paris Sud, Université Paris-Saclay, CNRS UMR 3348, 91401 Orsay, France; 3 Équipe Labellisée Ligue Contre le Cancer, 91401 Orsay, France; 4 INSERM U981, Gustave Roussy Cancer Campus, 94805 Villejuif, France; ramdane.guemiri@gmail.com; 5 Faculté de Médecine, Université Paris Sud, Université Paris-Saclay, 94270 Kremlin-Bicêtre, France

**Keywords:** alternative polyadenylation, intronic polyadenylation, silvestrol, translation, eIF4A, melanoma, immune checkpoints, STAT1

## Abstract

**Simple Summary:**

Immune checkpoints blockade has emerged as an effective approach to prevent immune escape of tumor cells, and constitutes a powerful anti-cancer therapeutic strategy. Regulation of the expression of genes encoding immune checkpoint inhibitors has thus become an increasingly important field of study. Beyond transcription, gene expression is regulated at several post-transcriptional levels including pre-mRNA 3′-end processing and mRNA translation. More specifically, the eIF4F translation initiation complex represents an important hub for oncogenic signaling in the etiology of different cancers. The eIF4A RNA helicase component of the eIF4F can be inhibited by the widely characterized small molecule inhibitor silvestrol. Here, we evaluated the effect of eIF4A inhibition with silvestrol on the translation of alternatively polyadenylated mRNAs in melanoma cell lines and activated T cells. We show that silvestrol can selectively inhibit the translation of alternatively polyadenylated isoforms of genes encoding key immune-related proteins.

**Abstract:**

Targeting the translation initiation complex eIF4F, which binds the 5′ cap of mRNAs, is a promising anti-cancer approach. Silvestrol, a small molecule inhibitor of eIF4A, the RNA helicase component of eIF4F, inhibits the translation of the mRNA encoding the signal transducer and activator of transcription 1 (STAT1) transcription factor, which, in turn, reduces the transcription of the gene encoding one of the major immune checkpoint proteins, i.e., programmed death ligand-1 (PD-L1) in melanoma cells. A large proportion of human genes produce multiple mRNAs differing in their 3′-ends through the use of alternative polyadenylation (APA) sites, which, when located in alternative last exons, can generate protein isoforms, as in the *STAT1* gene. Here, we provide evidence that the STAT1α, but not STAT1β protein isoform generated by APA, is required for silvestrol-dependent inhibition of PD-L1 expression in interferon-γ-treated melanoma cells. Using polysome profiling in activated T cells we find that, beyond STAT1, eIF4A inhibition downregulates the translation of some important immune-related mRNAs, such as the ones encoding TIM-3, LAG-3, IDO1, CD27 or CD137, but with little effect on the ones for BTLA and ADAR-1 and no effect on the ones encoding CTLA-4, PD-1 and CD40-L. We next apply RT-qPCR and 3′-seq (RNA-seq focused on mRNA 3′ ends) on polysomal RNAs to analyze in a high throughput manner the effect of eIF4A inhibition on the translation of APA isoforms. We identify about 150 genes, including TIM-3, LAG-3, AHNAK and SEMA4D, for which silvestrol differentially inhibits the translation of APA isoforms in T cells. It is therefore crucial to consider 3′-end mRNA heterogeneity in the understanding of the anti-tumor activities of eIF4A inhibitors.

## 1. Introduction

Immune checkpoint blockade is one of the most effective approaches to activate therapeutic antitumor immunity as tumors often use immune-checkpoint pathways as a major underlying mechanism of immune resistance. This involves immune receptors that negatively regulate antitumor adaptive T cell (T lymphocyte) responses, such as Cytotoxic T-lymphocyte-associated antigen 4 (CTLA-4) and programmed cell death protein 1 (PD-1) or its ligand PD-L1. Antibodies targeting these receptors are now widely used to treat a broad range of cancers. Although these new immunotherapies represent a huge improvement in the field of cancer therapies, early or late resistance emerge in the majority of the patients [1,2]. There is thus a high medical need to better understand the mechanisms underlying the control of immune checkpoint gene expression that is so far essentially described at the transcription level [3,4,5,6].

Following their transcription, most eukaryotic precursor messenger RNAs (pre-mRNAs) undergo a number of nuclear processing events including (i) a 5′ end capping reaction, (ii) splicing that is the removal of introns and subsequent ligation of exons, and (iii) a 3′-end RNA cleavage followed by addition of a polyadenylated tail at a polyadenylation site (pA site) on the pre-mRNA. The 3′-polyadenylated tail of mRNAs is necessary for their transport to the cytoplasm, their stability and translation [7,8,9,10]. Alternative polyadenylation (APA), which occurs in about two-thirds of human genes, is the alternative usage of distinct pA sites in genes [11]. APA can lead to the production of mRNAs with different lengths of their 3′ untranslated region (3′UTR) or with different protein coding capacities. In the latter case (called intronic polyadenylation; IPA), an alternative pA site located upstream of the last exon of the gene is used, leading to an alternative last exon (which may or may not be annotated), and the resulting alternatively polyadenylated mRNA isoform differs not only in its 3′UTR nature but also in its carboxy-terminal coding region [12,13,14].

Translational control has emerged as an important regulatory mechanism associated with many hallmarks of cancer. The eIF4F complex, composed of the 5′-cap-binding protein eIF4E, the RNA helicase eIF4A and the scaffolding protein eIF4G, is one of the most extensively studied RNA binding complexes involved in translational control in cancer [15,16,17,18]. eIF4F promotes the recruitment of the 40S ribosomal subunit to the cap. This recruitment is dependent on several features of the mRNA, including the level of RNA secondary structure in the 5′UTR, which is controlled by the unwinding activity of the eIF4A RNA helicase. Thus, specific mRNAs are more dependent on the eIF4F complex and eIF4A activity [19]. Among numerous small-molecule inhibitors of this complex reported to exert antitumor effects, silvestrol, a natural small-molecule selective inhibitor of eIF4A, is one of the most extensively studied [20,21,22]. eIF4A targeting by silvestrol selectively inhibits the translation of important oncogenic mRNAs containing a high level of secondary structure in their 5′UTR, hence exerting antitumoral effects [15,20,23,24,25,26].

We recently showed that, in interferon-γ-treated melanoma cells, translation of the signal transducer and activator of transcription 1 (STAT1) transcription factor is upregulated in an eIF4F-dependent manner, leading to transcriptional upregulation of *PD-L1* [16]. In fact, the *STAT1* gene has two alternative pA sites that generate a short transcript encoding the STAT1β protein and a long transcript encoding the most studied STAT1α protein. More generally, many genes were recently described to have alternatively polyadenylated mRNA variants in human immune cells [27]. With the exception of a few genes [28,29,30], the differential functions of protein isoforms encoded by alternatively polyadenylated mRNAs are poorly documented. Here, we evaluated the effect of inhibiting eIF4A with silvestrol on the translation of alternatively polyadenylated mRNA isoforms in both melanoma and T cells.

## 2. Materials and Methods

### 2.1. Cell Culture and siRNA Transfections

A375, SK-MEl-2, WM793, MCF-7, and MDA-MB-231 cells were grown in DMEM (Eurobio) containing 10% FBS (Pan Biotech, AidenBach, Germany) and L-Glutamine (Eurobio Scientific, Les Ulis, France) at 37 °C and 5% CO_2_. siRNA reverse transfections were carried out in 10 cm tissue culture dishes with Lipofectamine RNAiMAX (Thermo Scientific, Les Ulis, France). The siRNAs (Dharmacon, Cambridge, UK) were used at a final concentration of 30 nM; STAT1 α: TGTTATAGGTTGTTGGATA and STAT1 β: CAGAAGAGTGACATGTTTA) as per the manufacturer’s instructions in OptiMEM reduced serum media (Thermo Scientific). Isolated T cells from PBMCs (whole blood of healthy donors acquired from Établissement Français du Sang, Île de France) and Jurkat cells were cultured in RPMI (Eurobio Scientific, Les Ulis, France) supplemented with 10% (*v*/*v*) fetal bovine serum (PAN Biotech, Aidenbach, Germany), 2 mM L-glutamine (Eurobio Scientific, Les Ulis, France) and maintained at 37 °C in 5% CO_2_. They were stimulated with ImmunoCultTM Human CD3/CD28/CD2 T Cell Activator (StemCell #10990) and IL-2 (Peprotech 200-02) for 72 h. For protein analyses: 24 h after transfection, cells were treated with 10 nM or 30 nM silvestrol (MedChemExpress HY-13251, Sollentuna, Sweden). In addition, 48 h after transfection, cells were washed with PBS harvested on ice. For polysome experiments: Treatment with silvestrol was performed at a final concentration of 10 nM for 2 h.

### 2.2. Flow Cytometry Analysis

Cells were harvested by scraping on ice, treated with Fc Block (BD Biosciences, Rungis, France) in PBS/EDTA/BSA, washed and then incubated with the primary antibody human PD-L1 (Biolegend 329708, Paris, France) for 30 min. They were then incubated with PBS/EDTA/BSA containing Zombie NIR (live/dead discriminant) and then resuspended in 500 μL PBS/EDTA/BSA. An LSRII flow cytometer (BD Biosciences) was used for the acquisition of stained cells and the analysis of acquired data was performed using the FlowJo software. During acquisition of stained cells, debris (low FSC and SSC) was excluded and a selection of single cells that were negative for the live/dead discriminant was done by gating. An isotype negative control was used to eliminate a non-specific background signal according to the datasheet supplied with each antibody.

### 2.3. Western Blot

Cells were harvested on ice in PBS and centrifuged at 400× *g* for 5 min at 4 °C. The cell pellets were resuspended in RIPA Buffer containing phosphatase inhibitors and protease inhibitors (EDTA-free) (Thermo Scientific, Les Ulis, France). A dosage of the protein content in the cell lysates was performed using a bicinchoninic acid protein assay kit (Thermo Scientific, Les Ulis, France). Protein samples were then loaded onto denaturing NuPAGE gels (Life Technologies, Alfortville, France), resolved and transferred to a 0.45-mm nitrocellulose membrane (Bio-Rad, Marnes-la-Coquette, France). Blocking of the membranes was carried out in a buffer containing TBS, Tween-20 and 5% milk, following which they were incubated with the appropriate antibodies. Visualization of proteins was carried out using an ECL system (Bio-Rad). Relative density was calculated for quantitative analysis of band intensities using the ImageJ software (Java 1.8.0_172, imagej.nih.gov accessed on 19 January 2022, Bethesda, MD, USA).

The following primary antibodies were used: STAT1 (Cell Signaling Technology 14994) and SEMA4D (Thermofisher Scientific H00010507-M01).

### 2.4. Polysomal Fractionation and Profiling

A fractionation of subpolysomal and polysomal ribosome fractions was performed by sucrose density gradient centrifugation. The cells in culture were collected following a treatment with 100 μg/mL cycloheximide at 37 °C. They were harvested by scraping on ice in cold PBS containing 100 μg/mL cycloheximide, centrifuged at 400× *g* for 5 min and then resuspended in 400 μL of hypotonic buffer (5 mM Tris, pH 7.5, 1.5 mM KCl, 2.5 mM MgCl_2_, complete protease and phosphatase inhibitors) containing 0.5% Triton X-100, 0.5% sodium deoxycholate, 2 mM DTT, 400 U/mL RNaseOUT and 100 μg/mL cycloheximide. The lysates were loaded onto a 5–50% sucrose density gradient and centrifuged in an SW41 rotor at 38,000 rpm for 2 h at 4 °C. The polysomal profiles were monitored, and the fractions collected using a gradient fractionation system.

### 2.5. mRNA Preparation and Real-Time/Quantitative PCR

For the polysome profiling experiments, RNA extraction was performed using the TRIzol method (TRIzol-LS) from 250 μL of each fraction. The SuperScript IV Reverse Transcriptase (Thermo Scientific) kit was used for cDNA synthesis using random hexamer primers according to the manufacturer’s instructions. Equal volume of cDNA from each fraction was used to carry out the qRT-PCR experiments. For total RNA preparation, mRNA isolation was performed using TRIzol (Invitrogen, Paris, France) according to standard procedures. qRT-PCR was performed using the PowerUP qPCR Master Mix (Thermo Scientific) and SuperScript IV Reverse Transcriptase (Thermo Scientific) and was monitored on a Viia 7 System (Applied Biosystems, Paris, France). The HPRT gene was used to normalize the results in 2^−ΔΔCt^ analyses. The primer sequences of each cDNA were designed using Primer-BLAST (ncbi.nlm.nih.gov accessed on 19 January 2022) (Table S1).

### 2.6. 3′-Seq Experiments

3′-seq libraries were prepared with QuantSeq 3′ mRNA-Seq Library Prep Kit REV for Illumina (Lexogen, Vienna, Austria) using 500 ng of polysomal and input (cytosolic) RNAs (*n* = 3 for each condition) following manufacturer’s instructions. Purified libraries were quantified with Quant-iT Picogreen dsDNA kit (ThermoFisher Scientific) and run on Experion automated electrophoresis system (Bio-Rad). Pooled libraries were quantitated by qPCR (KAPA Library Quantification Kits Illumina Platforms, Roche), diluted to 12 pM, and subjected to single-end, 51 bp sequencing using the NovaSeq 2500 machine (Illumina).

### 2.7. 3′-Seq Bioinformatic Analysis

For all samples, raw reads were trimmed to remove uninformative nucleotides arising from primer sequences. Trimmed reads of 25 bp or more were aligned on the human reference genome (hg19) using Bowtie2 (version 2.2.5). [31] Mapping quality score (MAPQ) was determined and only reads with a score of 20 or more were selected (Samtools version 1.1) for downstream analysis. Clustering of reads was carried out along the genome using Bedtools (version 2.17.0) [32], allowing a maximum distance of 50 bp and a minimum number of 5 reads per peak. Peaks with a stretch of 6 consecutive adenosines (or 8 adenosines out of 9 nucleotides) within 50 bp downstream were filtered out, as they are likely due to internal priming of oligo-dT. Overlapping peaks from all samples of the conditions under comparison were merged to define a common set of genomic sequences corresponding to polyA sites. Location of peaks within genes was annotated using gene coordinates on the basis of overlapping Refseq transcripts with the same gene symbol. Peaks localized in the intronic region of a gene were classified as intronic polyA (IPA) peaks and those in the last exon of a gene were classified as LE peaks. Differential analyses between control and treated conditions were done with three independent biological replicates per condition. Comparing of the regulation of each IPA to the regulation of the gene’s last exon (taken as the sum of the peaks in this exon), we used the version 1.4.5 [33] and the following statistical model:

Y_ij_ = μ + L_i_ + C_j_ + (LC)_ij_ + E_ij_

where Y_ij_ is the normalized counts of peak i in biological condition j, μ is the mean, L_i_ is the peak localization (IPA or LE), C_j_ is the biological condition, (LC)_ij_ is the interaction between peak localization and biological condition, and E_ij_ is the residual. *p*-values and adjusted *p*-values (Benjamini–Hochberg) were calculated. Data with *p* < 0.05 are shown. The complete bioinformatics pipeline (3′-SMART package) explained in this paper is freely available at GitHub [34] and can be run through a configuration file and a simple command line. Annotated polyadenylation sites were retrieved from the Polya_DB 3 and PolyASite 2.0 databases [35,36].

### 2.8. Statistics

Statistical significance of the differences between experimental and control samples was assessed by an unpaired *t*-test and represented using GraphPad Prism (version 9.2.0), with significance achieved at *p* < 0.05.

## 3. Results

### 3.1. Functional Importance of APA-Generated STAT1 Protein Isoforms for PD-L1 Gene Expression

**Figure 1:**
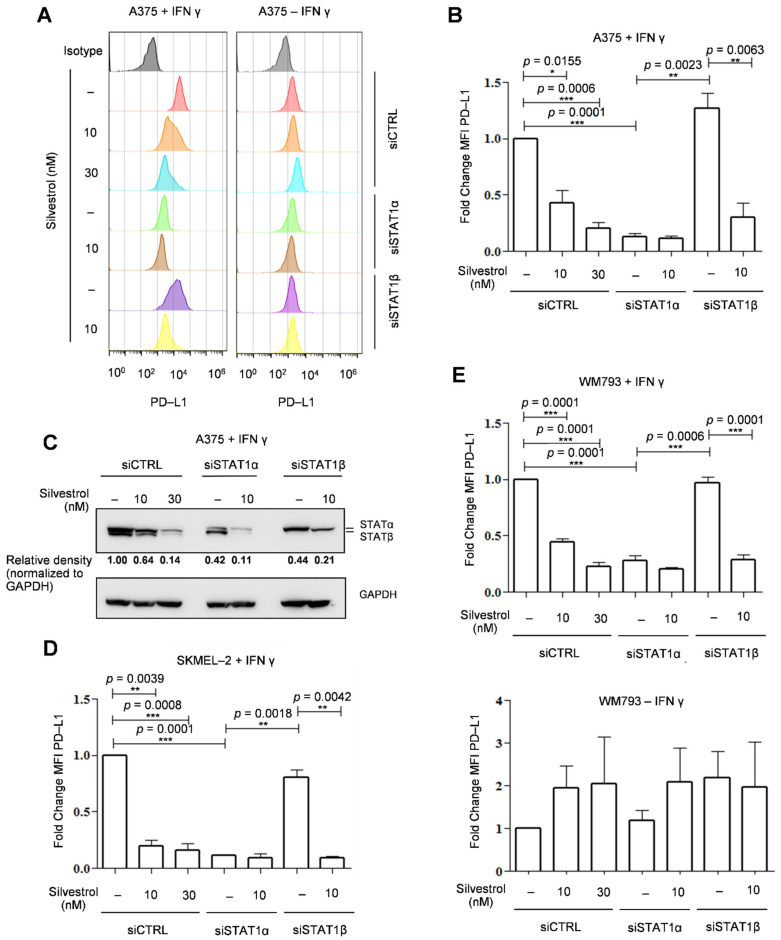
Functional importance of APA-generated STAT1 protein isoforms on PD-L1 gene expression. (**A**) PD-L1 was visualized by flow cytometry in A375 melanoma cells treated with IFN-γ (100 ng/mL) and silvestrol (10 nM or 30 nM); (**B**) PD-L1 mean fluorescence intensity (MFI) quantification in A375. The data are presented as the mean ± s.e.m. (*n* = 3 independent experiments). *p*-values were calculated using two-tailed unpaired *t*-test. Statistical analyses were performed for all data, *p*-values are indicated exclusively in the case of statistical significance; (* *p* ≤ 0.05, ** *p* ≤ 0.01, *** *p* ≤ 0.001) (**C**) Western blot analysis of the indicated proteins in A375. Quantification of STAT1 expression was performed by calculating the relative densities normalized to GAPDH levels (uncropped western blot original images see Figure S8). (**D**) same as (**B**) in SKMEL-2 (**E**) same as (**B**) in WM793 with (top) or without (bottom) IFN-γ.

In order to study the effect of the selective regulation of the two STAT1 protein isoforms on *PD-L1* expression, we depleted each of them separately by using specific siRNAs (Materials and Methods) in the BRAF V600E mutant A375 melanoma cell line. As previously shown [16], the presence of PD-L1 at the cell surface (determined by FACS analysis) was inhibited by silvestrol in interferon γ (IFN-γ) treated cells (Figure 1A,B). Cells were transfected with siRNAs that selectively depleted either the STAT1α or STAT1β isoform, as shown by Western blot with an antibody detecting both protein isoforms (Figure 1C, and Figures S1C and S8). In STATα-depleted cells, IFN-γ-induced PD-L1 expression was almost entirely lost; hence, no effect of silvestrol was observed on PD-L1 levels. In contrast, in STAT1β-depleted cells, IFN-γ-induced PD-L1 expression as well as its inhibition by silvestrol were similar to that observed in control cells (that is, cells transfected with a control siRNA; Figure 1A,B). Thus, IFN-γ-induced PD-L1 expression is controlled by STAT1α, and this isoform is necessary to observe silvestrol-dependent inhibition of PD-L1 expression. This was also validated in the NRAS Q61R mutant cell line SKMEL-2 (Figure 1D and Figure S1A) and in another BRAF V600E mutant cell line WM793 (Figure 1E and Figure S1B). However, STAT1α depletion had less effects on PD-L1 expression in IFN-γ-treated WM793 cells (Figure 1E, top), as compared to the two other tested cell lines. This can be explained by the fact that WM793 cells have higher basal (without IFN-γ) expression levels of PD-L1, which is approximately three times higher than A375 (Figure S2). In the absence of IFN-γ, silvestrol did not inhibit PD-L1 expression, and no significant effect of depleting STAT1α or β isoforms was observed (Figure 1E, bottom). In addition, in WM793 cells, in spite of a higher basal level expression of PD-L1, there was still an induction of its expression upon treatment with IFN-γ (Figures S1B and S2). We therefore looked into this regulation in two other cell lines, namely MDA-MB-231 and MCF-7 (Figure S3A,B), which are breast cancer cell lines with high basal expression levels of PD-L1 (Figure S2) [37]. In these cell lines, PD-L1 expression levels were not increased by IFN-γ and were not decreased by either silvestrol treatment or STAT1 isoform depletion (Figure S3A,B). Altogether, these results indicate that the STAT1α-dependent silvestrol inhibition of PD-L1 protein expression levels is strictly manifested upon PD-L1 induction by IFN-γ, which might be of importance in the context of immunotherapy.

### 3.2. Both STAT1 mRNA Isoforms Are Regulated by eIF4A Inhibition at the Translational Level

**Figure 2:**
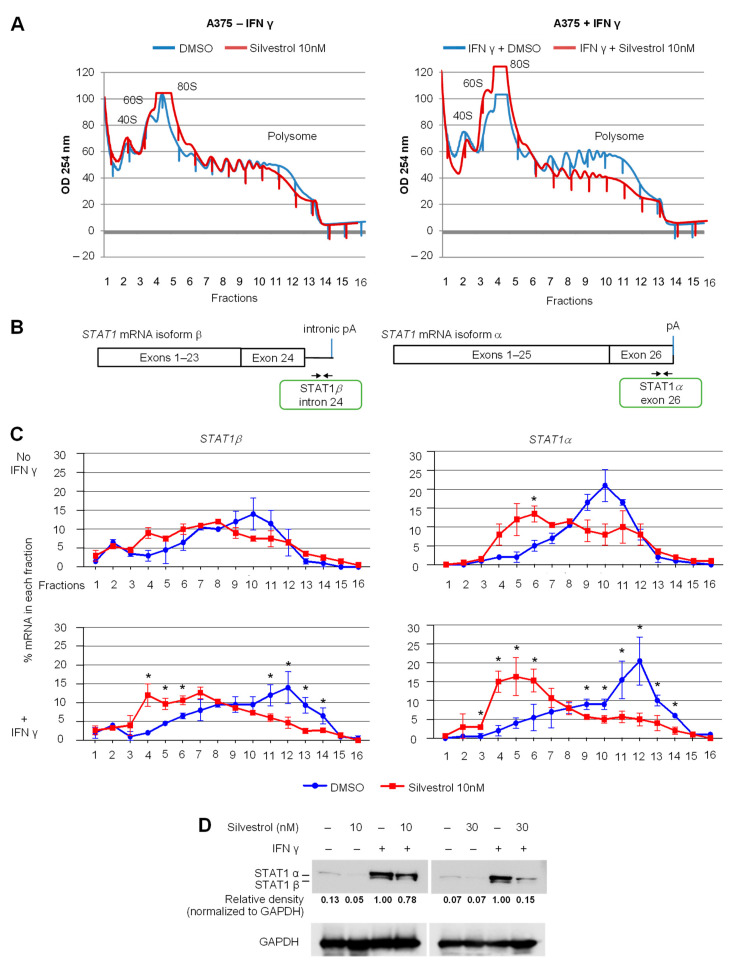
Both *STAT1* mRNA isoforms are regulated at the translational level by eIF4A inhibition. (**A**) Polysome profiles of A375 cells treated with IFN-γ for 24 h and silvestrol (10 nM) for 2 h. One representative profile from three independent experiments is shown; (**B**) primer design for RT-qPCR detection of *STAT1* mRNA isoforms; (**C**) percentage of transcripts for each *STAT1* APA isoform in each polysomal fraction obtained by sucrose-gradient ultracentrifugation was quantified by RT-qPCR (*n* = 3). *p*-values were calculated using two-tailed unpaired *t*-test (* *p* ≤ 0.05); (**D**) Western blot analysis to look into the expression of *STAT1* APA isoforms in A375 cells treated with IFN-γ for 24 h and silvestrol (10 nM or 30 nM) for 24 h. Quantification of STAT1 expression was performed by calculating the relative densities normalized to GAPDH levels. One representative blot from three independent experiments is shown (uncropped western blot original images see Figure S8).

In order to test the effect of silvestrol on the translation of *STAT1α* and *β* mRNA isoforms, we used polysome profiling in A375 cells treated with 10 nM silvestrol in the presence or not of IFN-γ. Polysome profiling uses sucrose-gradient separation of mRNAs based on their differential association with ribosomes (Figure 2A). The polysome profiles showed an overall inhibitory effect of silvestrol on translation as evidenced by a reduced polysome peak (Figure 2A). The translational status of a specific mRNA species can be inferred from its relative presence (detected by RT-qPCR) in heavier polysome fractions where it is bound to multiple ribosomes (i.e., actively translated mRNAs), lighter polysome fractions (less translated mRNAs), and subpolysomal fractions (untranslated mRNAs). The analysis of mRNAs encoding STAT1α and STAT1β was done by RT-qPCR using specific primers (Figure 2B and Table S1) and revealed that both mRNAs shifted from heavy polysome fractions to lighter polysome fractions upon silvestrol treatment, especially in IFN-γ-treated cells (Figure 2C). This indicates that silvestrol inhibits the translation of both *STAT1* mRNA isoforms in IFN-γ-treated cells. Consistently, the level of both STAT1 protein isoforms, analyzed by Western blot, was decreased by silvestrol in IFN-γ-treated cells (Figure 2D, Figures S4 and S8), while there was no change in the levels of *STAT1* mRNA isoforms in total cytosolic RNA (Figure S5).

### 3.3. eIF4A Inhibition Regulates the Translation of mRNAs Encoding Key Immune Checkpoint Proteins in Activated T Cells

**Figure 3:**
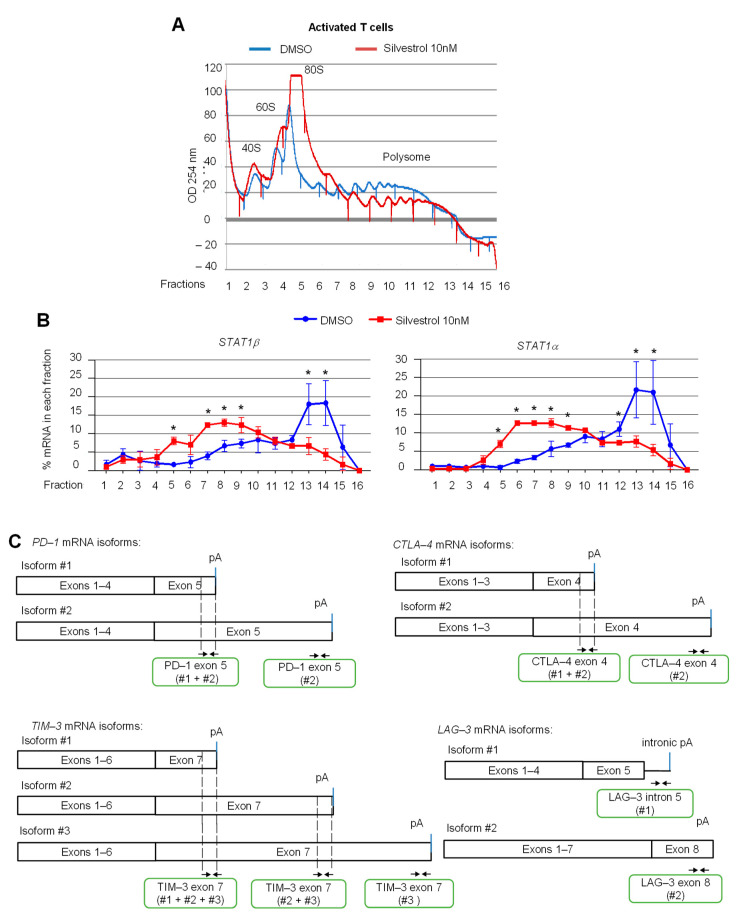

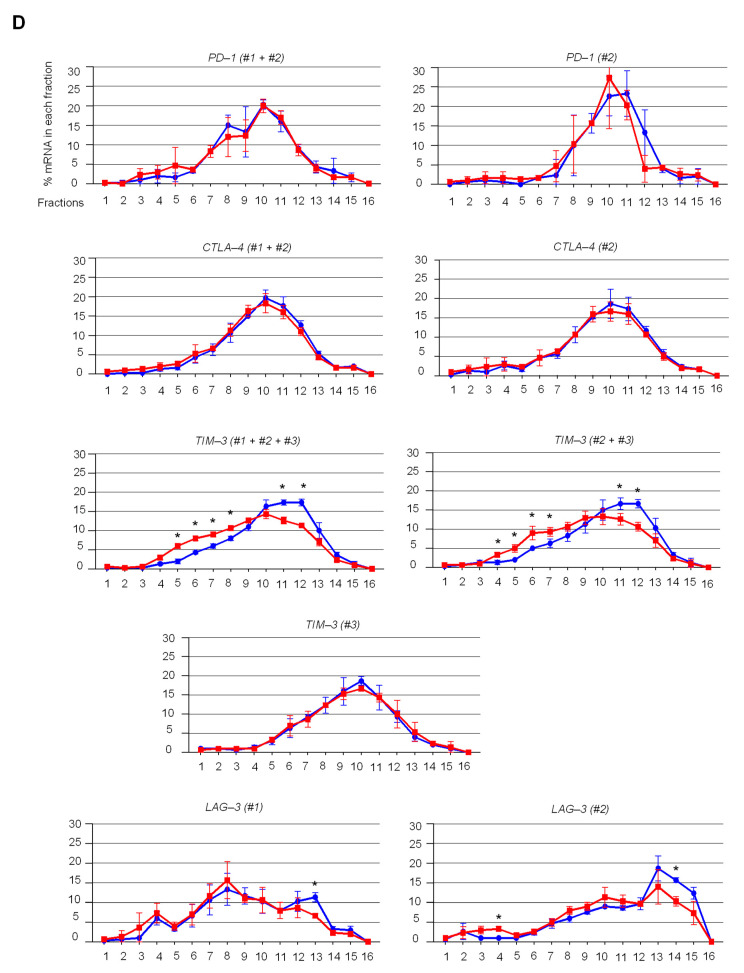
eIF4A inhibition regulates the translation of mRNAs encoding key immune checkpoint proteins such as TIM-3 and LAG-3, but not PD-1 and CTLA-4, in activated T cells. (**A**) polysome profiles of T cells stimulated for 72 h and treated with silvestrol (10 nM or 30 nM) for 2 h. One representative profile from three independent experiments is shown. (**B**) Percentage of transcripts for each *STAT1* APA isoform in each polysomal fraction obtained by sucrose-gradient ultracentrifugation of T cells was quantified by RT-qPCR (*n* = 3). *p*-values were calculated using two-tailed unpaired *t*-test (* *p* ≤ 0.05); (**C**) primer design for immune checkpoint gene mRNA isoforms; (**D**) percentage of transcripts for each APA isoform in each polysomal fraction obtained by sucrose-gradient ultracentrifugation was quantified by RT-qPCR (*n* = 3). An asterisk (*) was inserted for each fraction to indicate a statistically significant difference (*p* < 0.05).

Since STAT1 and major immune checkpoint proteins are also expressed on immune cells, the effect of silvestrol on the translation of mRNAs encoding STAT1 and key immune checkpoint proteins, such as PD-1, CTLA-4, TIM-3 and LAG-3, was examined using polysome profiling of human peripheral blood T cells isolated from healthy donors and stimulated with IL-2 and anti-CD3/anti-CD28 antibodies. The polysome profiles showed an overall inhibitory effect of silvestrol on translation in such activated T cells, as evidenced by a reduced polysome peak (Figure 3A). Both *STAT1* mRNAs isoforms shifted from heavy polysome fractions to lighter polysome fractions upon silvestrol treatment (Figure 3B), indicating that silvestrol inhibits the translation of both *STAT1* mRNA isoforms in activated T cells, as observed above in melanoma cells (Figure 2C).

Then, the translational status of alternatively polyadenylated mRNA isoforms encoding the key immune checkpoints PD-1, CTLA-4, TIM-3 (APA within the last exon) and LAG-3 (APA in alternative last exons) was investigated by RT-qPCR using specific primers (Figure 3C). We found that silvestrol inhibited the translation of *TIM-3* mRNA isoforms #1 and #2, of LAG-3 isoform #2 (albeit less efficiently), but had no effect on translation of *TIM-3* isoform #3 and all *PD-1* and *CTLA-4* mRNA isoforms, and had less effect on *LAG-3* isoform #1 (Figure 3D). These results prompted us to extend the analysis to a few more immune-related genes. We found that silvestrol inhibited the translation of both alternatively polyadenylated mRNA isoforms transcribed from the *CD137*, *CD27* and *IDO1* genes, while it had no effect on the translation of alternatively polyadenylated mRNA isoforms transcribed from the *CD40-L* gene, and little effect on *ADAR-1* and *BTLA* genes (Figure S6A,B). These results indicate that, beyond *STAT1*, several immune-related mRNAs (such as *TIM-3*, *LAG-3*, *IDO1*, *CD27*, *CD137*) are inhibited by silvestrol at the translation level. In addition, there are cases where silvestrol differentially inhibits translation of APA isoforms of the same gene (such as *TIM-3* and *LAG-3*).

### 3.4. eIF4A Inhibition Differentially Regulates the Translation of APA Isoforms in Several Immune-Related Genes

**Figure 4:**
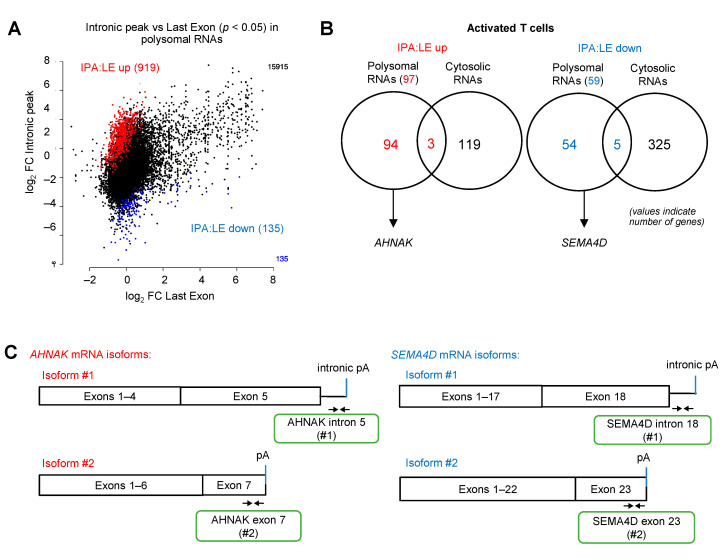

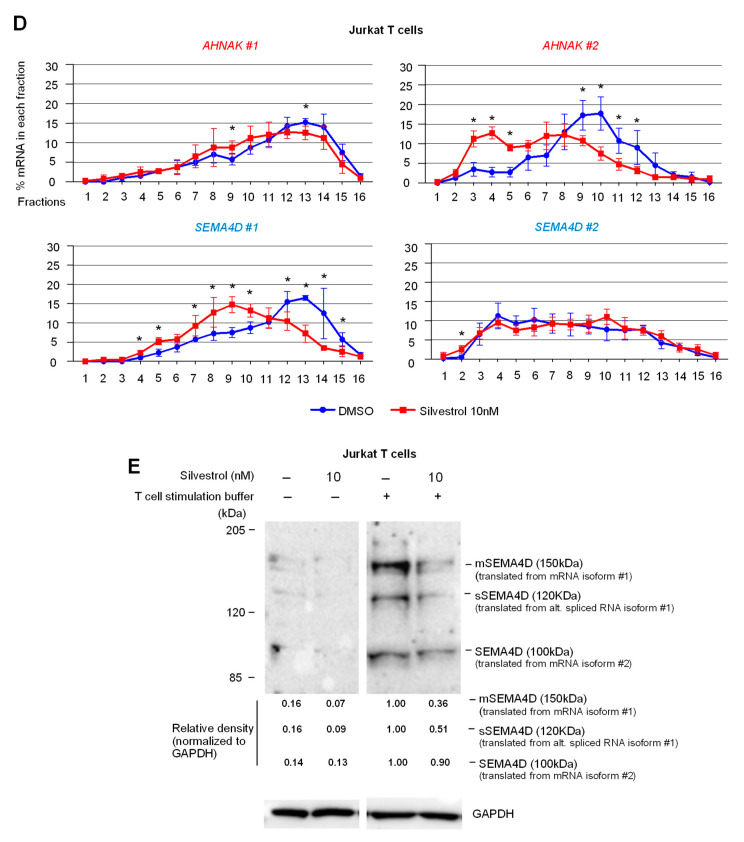
eIF4A inhibition regulates the translation of several immune-related alternatively polyadenylated mRNAs. (**A**) Regulation of intronic polyA (IPA) versus last exon (LE) isoforms in heavy polysome fractions following treatment with silvestrol (10 nM) for 2 h in CD8+ T cells from healthy donors. IPA sites that are significantly upregulated (IPA:LE up) or downregulated (IPA:LE down) by silvestrol relative to LE (*p*-adj < 0.1) are shown in red and blue, respectively; (**B**) number of genes with either up- or downregulation of IPA:LE isoform ratio by silvestrol in heavy polysome fractions or whole cytosol, as indicated; (**C**) primer design for candidate immune-related gene mRNA isoforms; (**D**) percentage of transcripts for each APA isoform in each polysomal fraction obtained by sucrose-gradient ultracentrifugation was quantified by RT-qPCR in Jurkat cells (*n* = 3). *p*-values were calculated using two-tailed unpaired *t*-test (* *p* ≤ 0.05); (**E**) Western blot analysis to look into the expression of SEMA4D APA isoforms in Jurkat cells without or with stimulation for 72 h including silvestrol (10 nM) treatment for 24 h. Quantification of SEMA4D expression was performed by calculating the relative densities normalized to GAPDH levels. One representative blot from three independent experiments is shown (uncropped western blot original images see Figure S8).

Our finding that silvestrol differentially inhibited the translation of the alternatively polyadenylated isoforms of the *TIM-3* and *LAG-3* genes prompted us to extend this observation in a high-throughput manner. We used a 3′-seq approach that consists of targeted sequencing of the 3′-end of polyadenylated transcripts upstream of the pA tail. We carried out 3′-seq on polysomal RNA of activated T cells treated with either silvestrol or vehicle for 2 h. We found that silvestrol had a significant effect on the ratio of (Intronic Polyadenylation) IPA to (Last Exon) LE isoforms in polysomes for 1054 genes (*p* < 0.05; Figure 4A). For 919 genes, the polysomal IPA:LE isoform ratio was increased by silvestrol (IPA:LE up), suggesting that silvestrol had more inhibitory effect on the translation of the LE isoform compared to the IPA isoform. For 135 genes, silvestrol had more translation inhibitory effect on the IPA than the LE mRNA isoform (IPA:LE down; Figure 4A). We then concentrated our analysis on the more abundant IPA isoforms, i.e., at least 5% of the abundance of last exon “LE” isoform from the same gene (Figure 4B and Table S2). When analyzing polysomal RNAs, we identified 97 genes for which the IPA:LE ratio was increased and 59 genes for which the ratio was decreased by silvestrol. Importantly, these changes in IPA:LE ratio were only observed in polysomal RNA, but not in cytosolic RNA, for 94 (97%) of the 97 genes in the “IPA:LE up” group and for 54 (92%) of the 59 genes in the “IPA:LE down” group (Figure 4B). This indicated that, in these genes, silvestrol changes the relative translatability of the IPA and LE mRNA isoforms, without changing their relative abundance.

To validate these regulation events, we focused on two genes, *AHNAK* (one of the 94 “IPA:LE up” candidates) and *SEMA4D* (one of the 54 “IPA:LE down” candidates) that have important immune functions. We selected these genes because they are relevant to T cell function. Indeed, AHNAK mediates calcium entry essential for cytolytic activity following T cell receptor (TCR) engagement of cytotoxic CD8+ T lymphocytes [38,39]. This AHNAK-dependent calcium signaling is also upregulated during CD4+ T cell activation [40,41] and a knockout of AHNAK inhibits T cell responses and the secretion of IFN-γ and IL-4 cytokines [42]. SEMA4D regulates cell proliferation by interacting with CD72 [43,44]. It downregulates the differentiation of regulatory T (Treg) cells by inhibiting Foxp3 expression [45]. In addition, antibody blockade of SEMA4D increases the efficiency of anti-PD1 and anti-CTLA4 immunotherapies [46,47].

Primers were designed to quantitate alternatively polyadenylated mRNA isoforms of AHNAK and SEMA4D by RT-qPCR on polysome fractions of the Jurkat T-lymphocyte cell line treated either with silvestrol or vehicle (Figure 4C). We confirmed that silvestrol inhibited the translation of the *AHNAK* LE (#2) more efficiently than IPA (#1) isoform, and of the *SEMA4D* IPA (#1) than LE (#2) isoform (Figure 4D). Consistent with these findings, the levels of SEMA4D protein isoforms, for which antibodies were available, were differentially regulated by silvestrol in activated Jurkat T cells (Figure 4E). Whereas both SEMA4D membrane-bound and soluble protein isoforms translated from the IPA isoform were decreased in silvestrol-treated cells, the smaller protein isoform translated from the LE isoform was not decreased (Figure 4E and Figure S8).

Additional candidate genes from either category, namely *POU6F1*, *HPS3* and *DENND1A* (IPA:LE up) and *FBXO11* and *DIP2A* (IPA:LE down) were also examined (Figure S7A,B). The translation of LE isoforms of “IPA:LE up” candidates and of IPA isoforms of “IPA:LE down” candidates were more strongly inhibited by silvestrol compared to the other alternatively polyadenylated mRNA isoform for each gene (Figure S7B). Altogether, these data identify genes, for which silvestrol selectively inhibits the translation of specific APA isoforms.

## 4. Discussion

Our findings provide several new insights into the effects of eIF4A inhibition on mRNA translation in the context of cancer.

We previously demonstrated that the expression of PD-L1 in cancer cells was decreased by silvestrol, but the effect was indirect and mediated by translation regulation of STAT1, a transcriptional regulator of PD-L1 [16]. In fact, two protein isoforms of STAT1 are encoded from two alternatively polyadenylated mRNA isoforms. We extended the conclusions from these findings by showing that the silvestrol-mediated inhibition of PD-L1 expression is dependent on STAT1α, but not STAT1β. This illustrates the relevance of alternative polyadenylation in generating protein isoforms with different functions. Beyond STAT1, there are a few examples illustrating the functional relevance of alternative polyadenylation in T cells. The T-cell surface glycoprotein CD5 is regulated by alternative polyadenylation during human T lymphocyte activation. Preferential 3′UTR shortening of CD5 mRNA upon T cell activation, due to proximal pA site usage, leads to higher CD5 expression and determines cellular outcomes of survival or apoptosis [48]. Another example of regulation of alternatively polyadenylated transcripts following T cell activation relates to CELF1/CUGBP1 target transcripts. A number of CELF1 regulated transcripts involved in cell division are generated with shorter 3′ ends to promote the exclusion of CELF1 binding sites, thereby upregulating their expression following stimulation of T cells [49].

Genome-wide studies showed that T-cell activation is associated with a selective usage of proximal pA sites, leading to a preferential increase in the expression of mRNAs with shorter 3′UTRs [50,51]. However, in most genes, this preferential shortening of the 3′UTR is not associated with increased protein production [50,52]. Here, by combining RNA analysis (through 3′-seq or RT-qPCR) and polysome profiling, we provide evidence that silvestrol differentially inhibits the translation of mRNAs with different 3′-ends produced through alternative polyadenylation. The main determinant of an mRNA for its dependence on the eIF4A RNA helicase is the level of stable secondary structures [53] or more complex G-quadruplex structures [54] within the 5′ UTR of mRNAs. It is possible that the 3′UTR of an mRNA influences 5′UTR-based mechanisms when the UTRs are in close proximity and that this makes an mRNA sensitive to silvestrol inhibition for certain 5′UTR-3′UTR combinations. Such proximity between the 5′UTR and 3′UTR has, for instance, been observed by electron microscopy on the membrane of the endoplasmic reticulum, where the great majority of polysomes have a circular shape [55,56]. It is also possible that the 3′UTR is involved in mRNA localization in specific subcellular regions that are more prone to silvestrol inhibition. It is very well admitted that 3′UTRs influence mRNA localization to very specialized cellular sites such as dendrites or synapses in neuronal cells [57,58]. mRNA localization is dependent on the repertoire of 3′UTR-bound RNA binding proteins. For instance, the TIS11B RNA binding protein interacts with specific 3′UTR-containing mRNAs to form membrane-less organelles called TIS granules intertwined with the endoplasmic reticulum [58]. Interestingly, several membrane protein-encoding mRNAs, including PD-L1, predominantly localize to TIS granules [58]. In addition, 3′UTRs are determinants of mRNA localization in stress granules [59,60]. It has been shown that inhibitory immune checkpoint mRNAs (including TIM-3 and LAG-3) require microtubule-dependent stress granule dynamics [61]. All these 3′UTR-dependent mechanisms should be carefully tested in the context of the 3′UTR-dependent inhibition of translation by silvestrol.

Finally, silvestrol is shown here for the first time to inhibit the translation of mRNAs encoding key immune checkpoint inhibitors (e.g., TIM-3 and LAG-3) in T cells. Considering the promising results achieved with inhibitors of LAG-3 [62] and TIM-3 [63], and the fact that eIF4A inhibitors are currently tested in clinical trials, it will be interesting to test combinations of eIF4A inhibitors with either TIM-3 or LAG-3 inhibitors. In this context, it will be essential to consider the 3′UTR-dependent inhibition of translation by eIF4A inhibitors to better understand the potential beneficial effect of these treatment combinations.

## 5. Conclusions

Our findings reveal that inhibiting the eIF4A RNA helicase component of the eIF4F translation initiation complex with silvestrol differentially inhibits alternatively polyadenylated mRNA isoforms of certain immune-related genes like *TIM-3*, *LAG-3*, *AHNAK* and *SEMA4D*, among others. They have important implications for treatment of human cancers with eIF4A inhibitors while some of them are entering into clinical trials. Investigating the impact of eIF4A targeting on the translation of alternatively polyadenylated mRNA isoforms of the same gene may contribute to the understanding of anti-cancer activities of eIF4A inhibitors.

## Data Availability

The 3′-seq data presented in this study are openly available in the Gene Expression Omnibus repository (GEO) under accession number GSE193966 (token: cdkviuggdjoldin). Computer Code and Software: The complete bioinformatics pipeline 3′-SMART can be freely downloaded at GitHub (https://github.com/InstitutCurie/3-SMART, accessed on 19 January 2022).

## Supplementary Materials

The following supporting information can be downloaded at: https://www.mdpi.com/article/10.3390/cancers14051177/s1, Figure S1: (A) PD-L1 was visualized by flow cytometry in SK-MEL-2 cells treated with IFN-γ (100 ng/mL) and silvestrol (10 nM or 30 nM). (B) PD-L1 was visualized by flow cytometry in WM793 cells treated with or without IFN-γ (100 ng/mL) and silvestrol (10 nM or 30 nM). (C) Western blot analysis to look into the effect of silencing either STAT1 isoform in A375 cells treated with or without IFN-γ for 24 h and silvestrol (10 nM or 30 nM) for 24 h. Quantification of STAT1 expression was performed by calculating the relative densities normalized to GAPDH levels. One representative blot from three independent experiments is shown; Figure S2: PD-L1 was visualized by flow cytometry in A375, WM793, MCF-7 and MDA-MB-231 cells treated without or with IFN-γ (100 ng/mL). Bottom: PD-L1 mean fluorescence intensity (MFI) quantification. The data are presented as the mean ± s.e.m. (*n* = 3 independent experiments). *p*-values were calculated using a two-tailed unpaired *t*-test; Figure S3: Left: PD-L1 was visualized by flow cytometry in (A) MCF-7 and (B) MDA-MB-231 cell lines treated with or without IFN-γ (100 ng/mL) and silvestrol (10 nM or 30 nM). Right: PD-L1 mean fluorescence intensity (MFI) quantification. The data are presented as the mean ± s.e.m. (*n* = 3 independent experiments). *p*-values were calculated using a two-tailed unpaired *t*-test. Bottom, Western blot analysis of the indicated proteins; Figure S4: Western blot analysis to look into the expression of *STAT1* APA isoforms in SK-MEL-2 and WM793 cells treated with IFN-γ for 24 h and silvestrol (10 nM or 30 nM) for 24 h. Quantification of STAT1 expression was performed by calculating the relative densities normalized to GAPDH levels. One representative blot from three independent experiments is shown; Figure S5: Relative mRNA expression of total and APA isoforms of *STAT1* and of *TBP* (control) in total (non-fractionated) lysates of A375 (*n* = 3). A375 cells were treated without or with IFN-γ for 24 h and silvestrol (10 nM or 30 nM) for 24 h; Figure S6: (A) Primer design for immune-related gene mRNA isoforms. (B) Percentage of transcripts for each APA isoform in each polysomal fraction obtained by sucrose-gradient ultracentrifugation was quantified by RT-qPCR (*n* = 3). *p*-values were calculated using two-tailed unpaired *t*-test (* *p* ≤ 0.05); Figure S7: (A) Primer design for immune-related gene mRNA isoforms of IPA:LE UP (red) and IPA:LE DOWN (blue) candidates. (B) Percentage of transcripts for each APA isoform in each polysomal fraction obtained by sucrose-gradient ultracentrifugation was quantified by RT-qPCR in Jurkat cells (n = 3). *p*-values were calculated using two-tailed unpaired *t*-test (* *p* ≤ 0.05); Figure S8: Uncropped Western blot images; Figure S8: Uncropped Western Blot original images; Table S1: Primer sequences used for RT-qPCR analysis of all mRNAs investigated in this paper; Table S2: List of genes with upregulated and downregulated IPA:LE isoform ratio, as identified from 3′ Sequencing analysis in heavy polysomes and whole cytosol. Columns A–C, coordinates of IPA peaks (hg19). Column D, IPA peak number in our analysis. Columns E–F and I, gene coordinates. Column G, gene symbol (please note that some genes contain several regulated IPA peaks). Column H, RefSeq information on coding (NM) or noncoding (NR) status of gene transcripts. Columns N–Y: Number of reads in each sample either in IPA peak (IPA) or in the gene’s last exon (LE). Biological replicates are indicated (n1 to n3). Silv, Silvestrol. HP, heavy polysomes. CYTO, whole cytosol. Column Z, abundance of IPA peak relative to last exon (in %). Columns AC and AD, hg19 coordinates of annotated polyA site(s) in published databases [35,36] overlapping with the regulated IPA peak.
