# Supplementary material for: Differential Effects on the Translation of Immune-Related Alternatively Polyadenylated mRNAs in Melanoma and T Cells by eIF4A Inhibition
Source: Cancers (Basel). 2022 Feb 24;14(5):1177. doi: 10.3390/cancers14051177 (PMC8909304; doi:10.3390/cancers14051177)
Supplement: Supplementary file 1 [file cancers-14-01177-s001.zip › cancers-1584621-supplementary.pdf]

cancers

MDPI

Supplementary Materials

Differential Effects on the Translation of Immune-Related Alternatively Polyadenylated mRNAs in Melanoma and T Cells by eIF4A Inhibition

Biswendu Biswas, Ramdane Guemiri, Mandy Cadix, Céline M. Labbé, Alina Chakraborty, Martin Dutertre, Caroline Robert and Stéphan Vagner

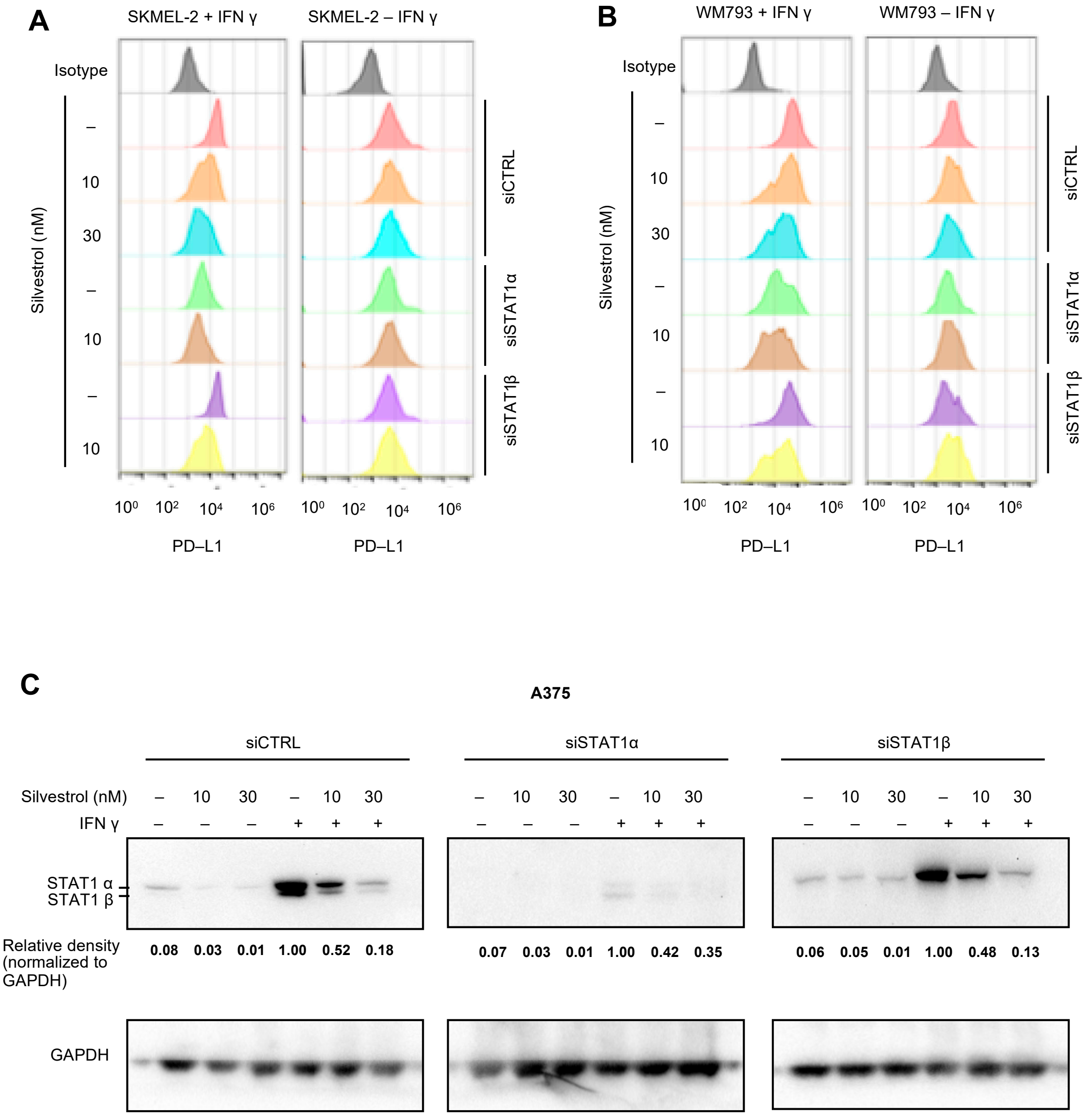

Figure S1. (A) PD-L1 was visualized by flow cytometry in SK-MEL-2 cells treated with IFN-γ (100 ng/mL) and silvestrol (10 nM or 30 nM). (B) PD-L1 was visualized by flow cytometry in WM793 cells treated with or without IFN-γ (100 ng/mL) and silvestrol (10 nM or 30 nM). (C) Western blot

analysis to look into the effect of silencing either STAT1 isoform in A375 cells treated with or without IFN-γ for 24 h and silvestrol (10 nM or 30 nM) for 24 h. Quantification of STAT1 expression was performed by calculating the relative densities normalized to GAPDH levels. One representative blot from three independent experiments is shown.

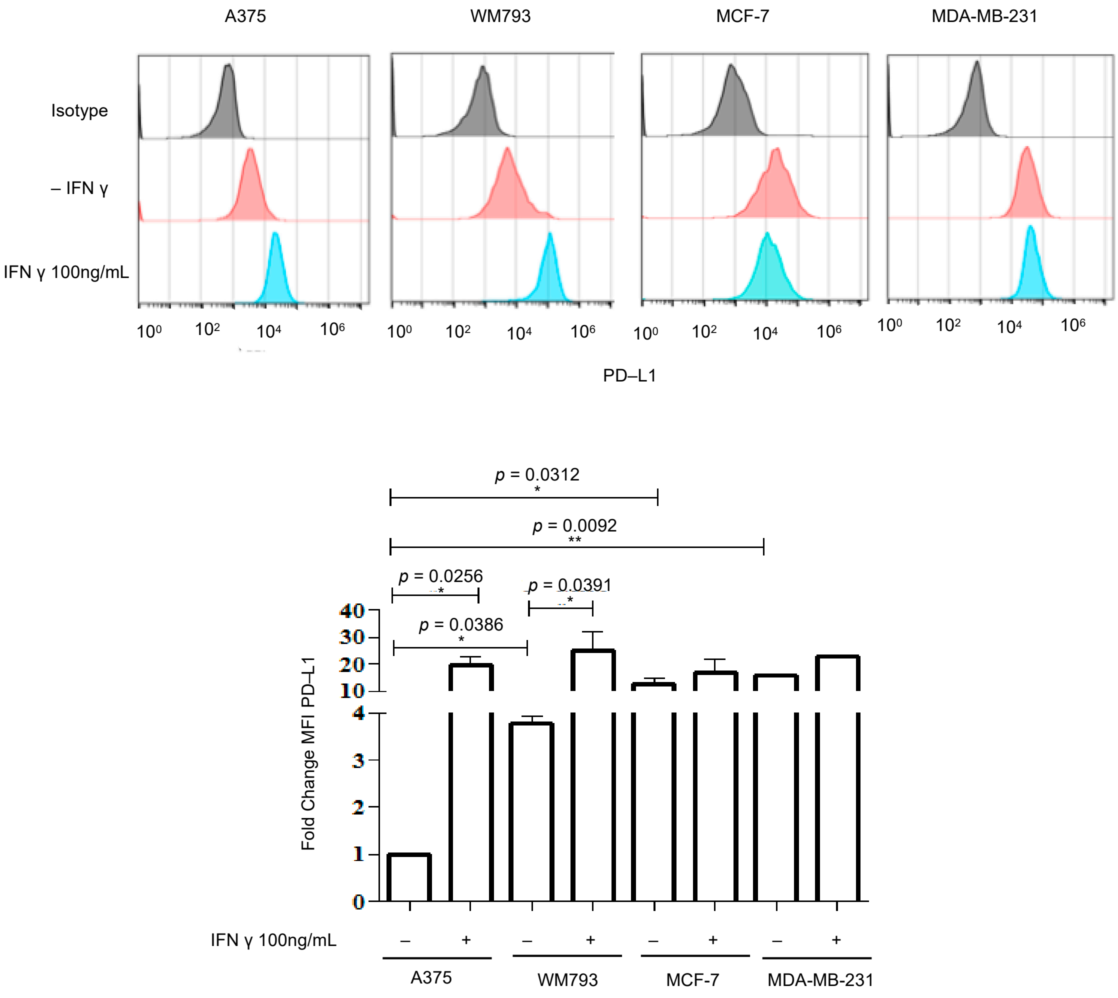

Figure S2. PD-L1 was visualized by flow cytometry in A375, WM793, MCF-7 and MDA-MB-231 cells treated without or with IFN-γ (100 ng/mL). Bottom: PD-L1 mean fluorescence intensity (MFI) quantification. The data are presented as the mean ± s.e.m. (n = 3 independent experiments). p-values were calculated using a two-tailed unpaired t-test.

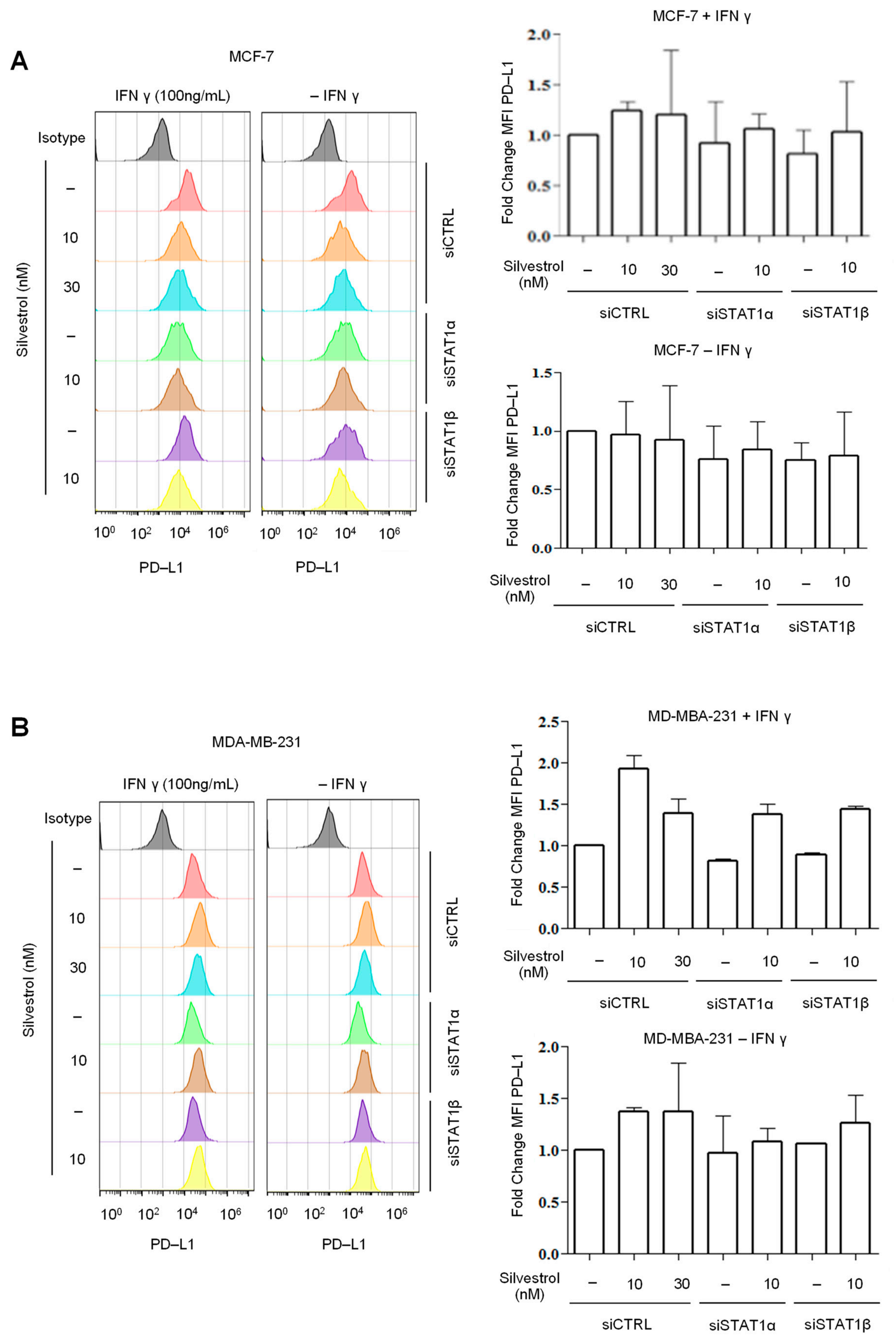

Figure S3. Left: PD-L1 was visualized by flow cytometry in (A) MCF-7 and (B) MDA-MB-231 cell lines treated with or without IFN-γ (100 ng/mL) and silvestrol (10 nM or 30 nM). Right: PD-L1 mean

fluorescence intensity (MFI) quantification. The data are presented as the mean ± s.e.m. (n = 3 independent experiments). p-values were calculated using a two-tailed unpaired t-test. Bottom, Western blot analysis of the indicated proteins.

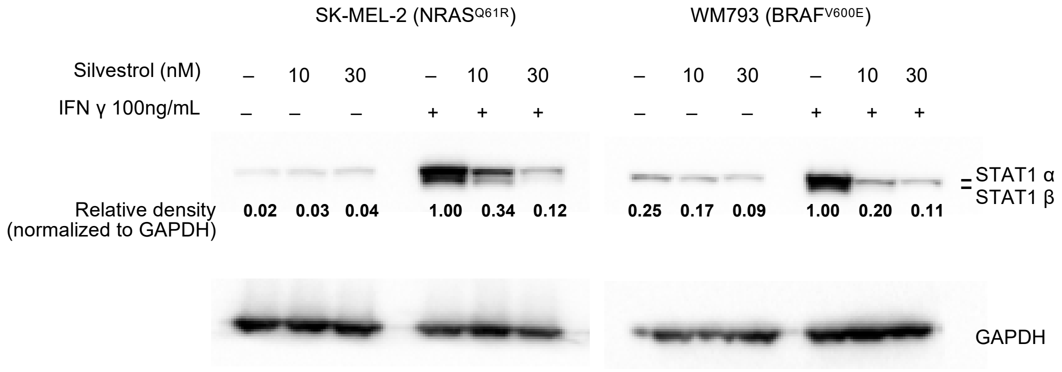

Figure S4. Western blot analysis to look into the expression of *STAT1* APA isoforms in SK-MEL-2 and WM793 cells treated with IFN-γ for 24 h and silvestrol (10 nM or 30 nM) for 24 h. Quantification of STAT1 expression was performed by calculating the relative densities normalized to GAPDH levels. One representative blot from three independent experiments is shown.

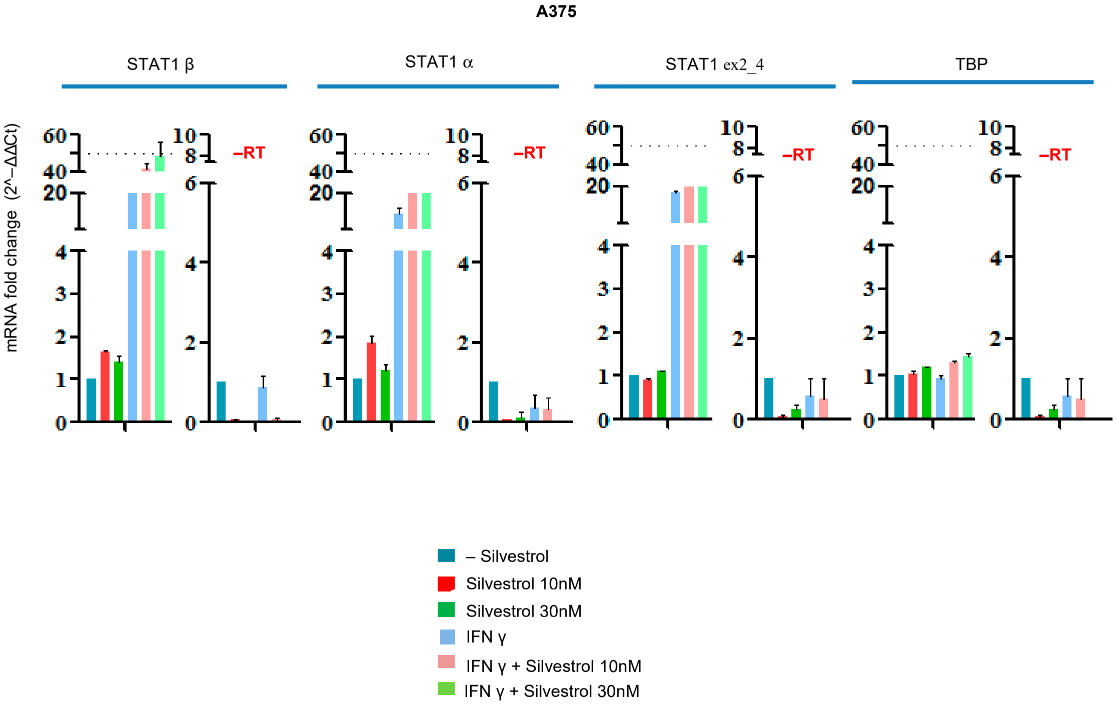

Figure S5. Relative mRNA expression of total and APA isoforms of *STAT1* and of *TBP* (control) in total (non-fractionated) lysates of A375 (n = 3). A375 cells were treated without or with IFN-γ for 24 h and silvestrol (10 nM or 30 nM) for 24 h.

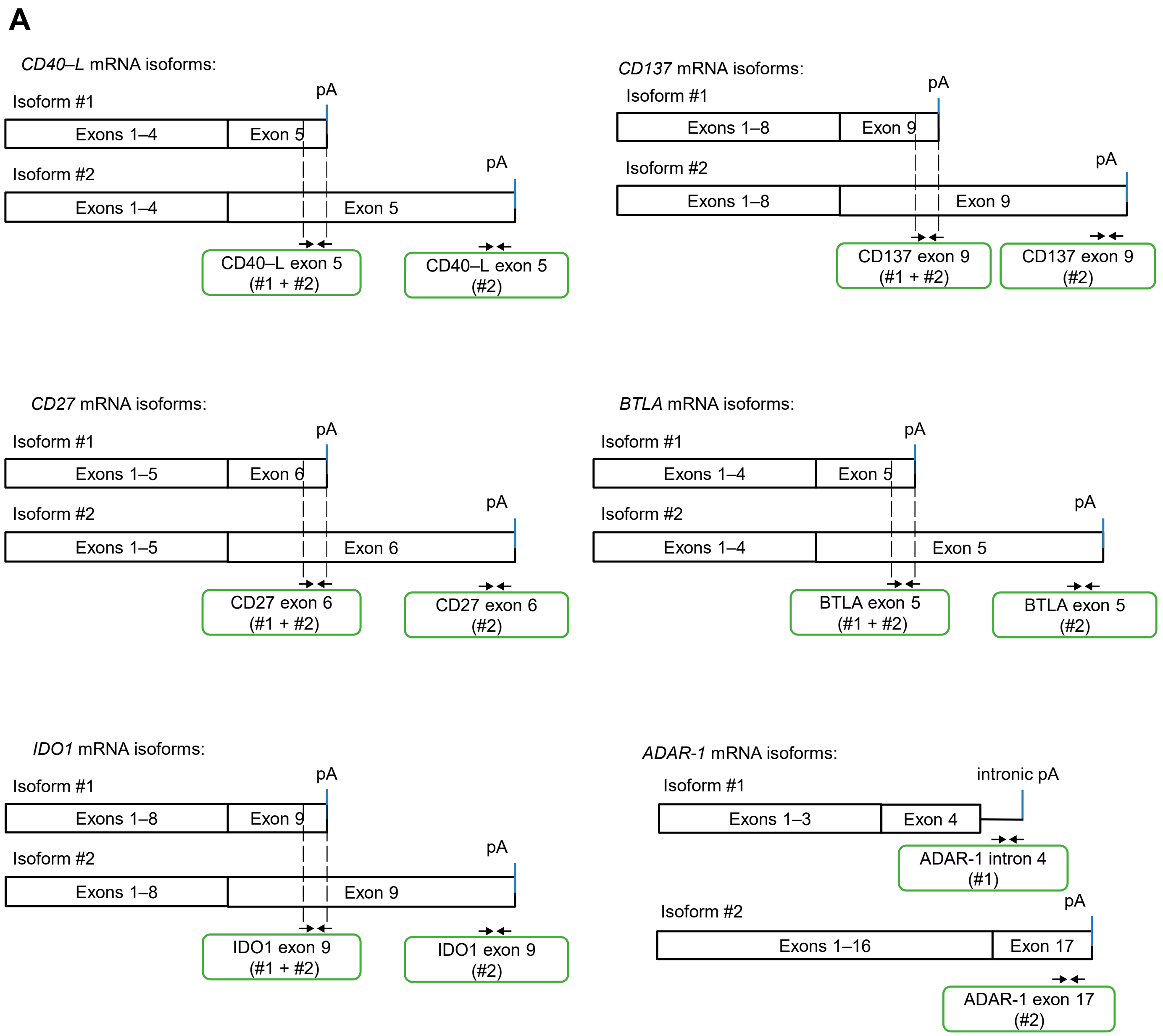
A
CD40–L mRNA isoforms:
Isoform #1
pA
Exons 1–4
Exon 5
Isoform #2
pA
Exons 1–4
Exon 5
CD40–L exon 5 (#1 + #2)
CD40–L exon 5 (#2)
CD137 mRNA isoforms:
Isoform #1
pA
Exons 1–8
Exon 9
Isoform #2
pA
Exons 1–8
Exon 9
CD137 exon 9 (#1 + #2)
CD137 exon 9 (#2)
CD27 mRNA isoforms:
Isoform #1
pA
Exons 1–5
Exon 6
Isoform #2
pA
Exons 1–5
Exon 6
CD27 exon 6 (#1 + #2)
CD27 exon 6 (#2)
BTLA mRNA isoforms:
Isoform #1
pA
Exons 1–4
Exon 5
Isoform #2
pA
Exons 1–4
Exon 5
BTLA exon 5 (#1 + #2)
BTLA exon 5 (#2)
IDO1 mRNA isoforms:
Isoform #1
pA
Exons 1–8
Exon 9
Isoform #2
pA
Exons 1–8
Exon 9
IDO1 exon 9 (#1 + #2)
IDO1 exon 9 (#2)
ADAR-1 mRNA isoforms:
Isoform #1
intronic pA
Exons 1–3
Exon 4
ADAR-1 intron 4 (#1)
Isoform #2
pA
Exons 1–16
Exon 17
ADAR-1 exon 17 (#2)

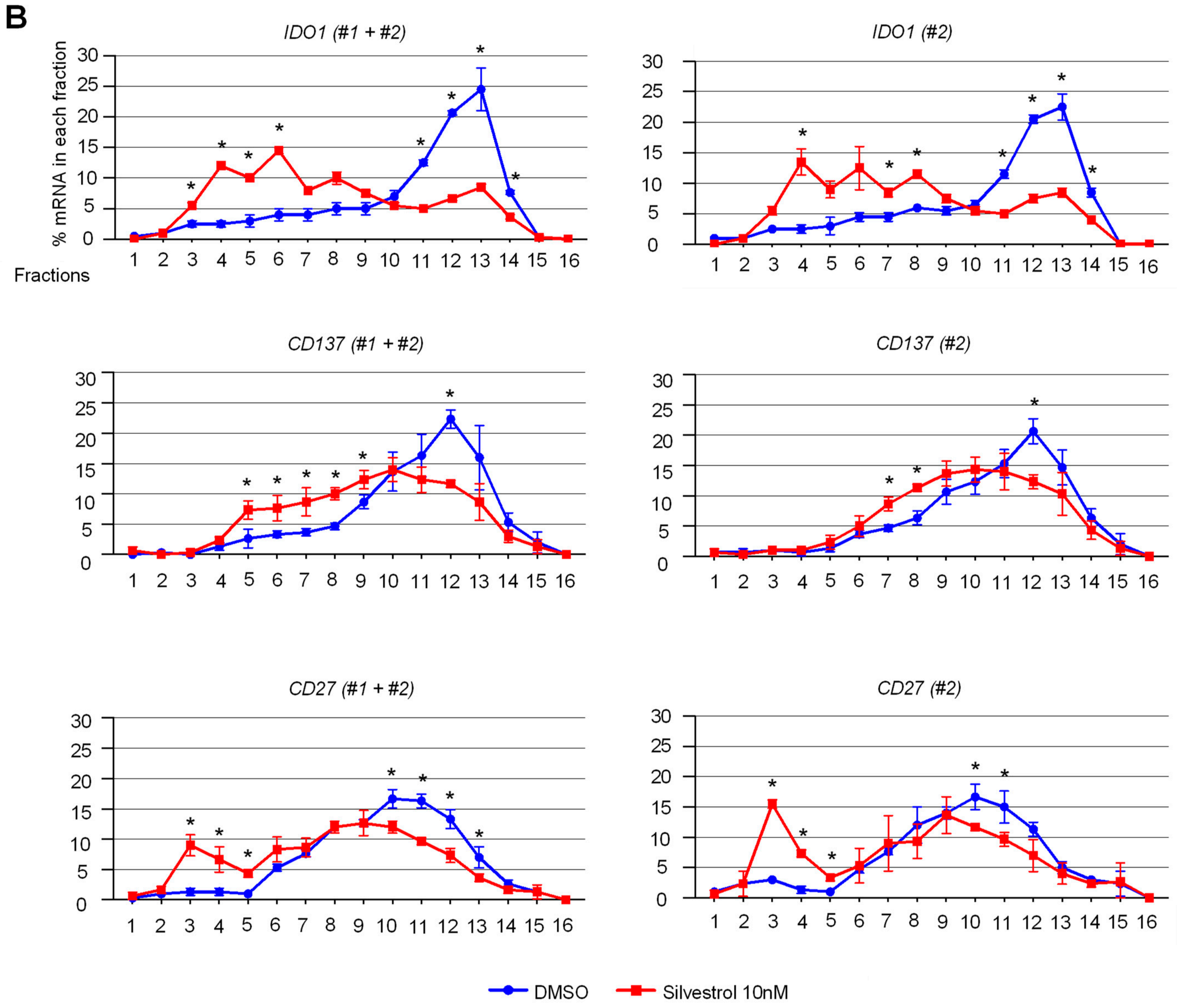

B
IDO1 (#1 + #2)
IDO1 (#2)
CD137 (#1 + #2)
CD137 (#2)
CD27 (#1 + #2)
CD27 (#2)
% mRNA in each fraction
Fractions
DMSO
Silvestrol 10nM

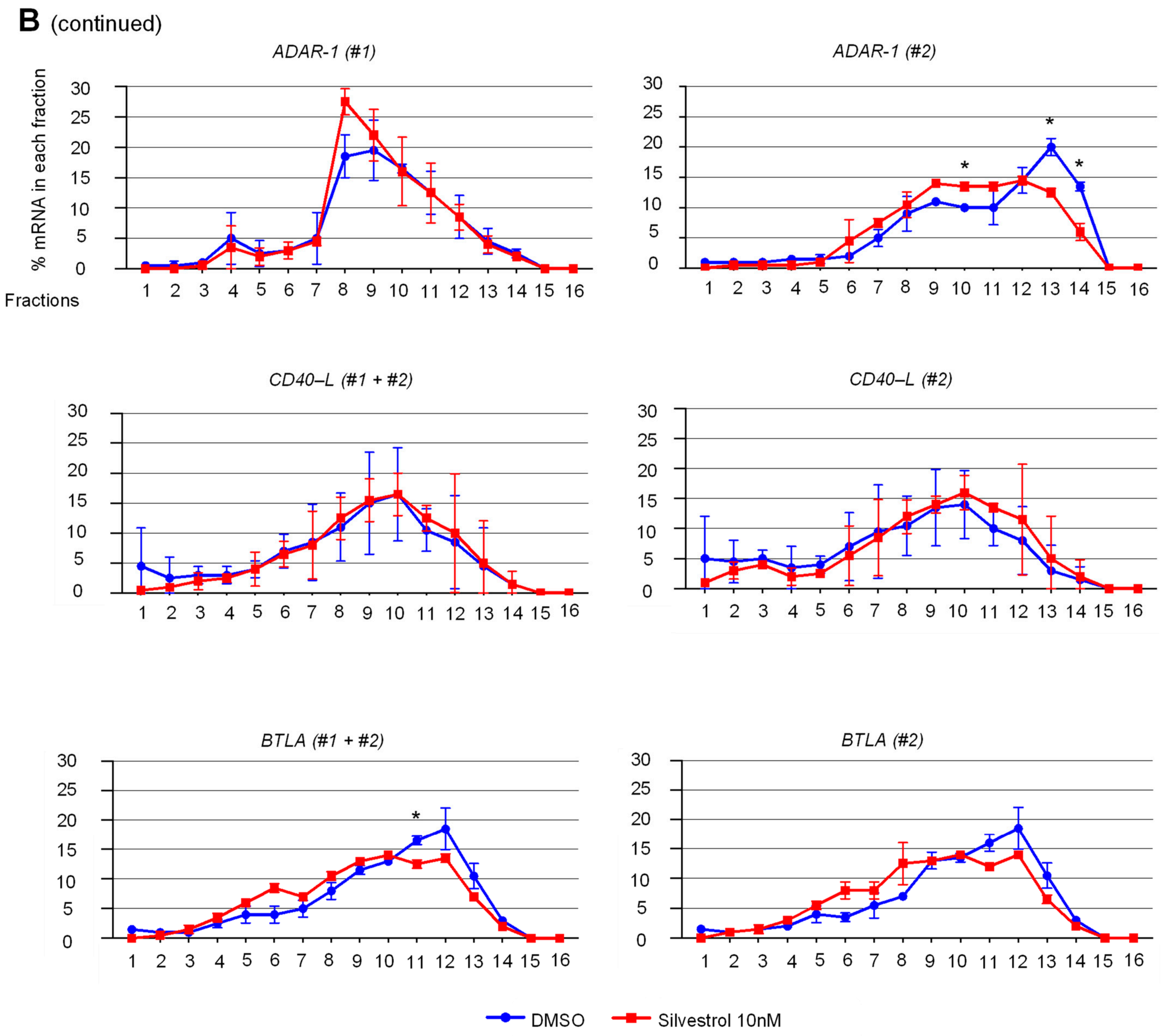

Figure S6. (A) Primer design for immune-related gene mRNA isoforms. (B) Percentage of transcripts for each APA isoform in each polysomal fraction obtained by sucrose-gradient ultracentrifugation was quantified by RT-qPCR (n = 3).

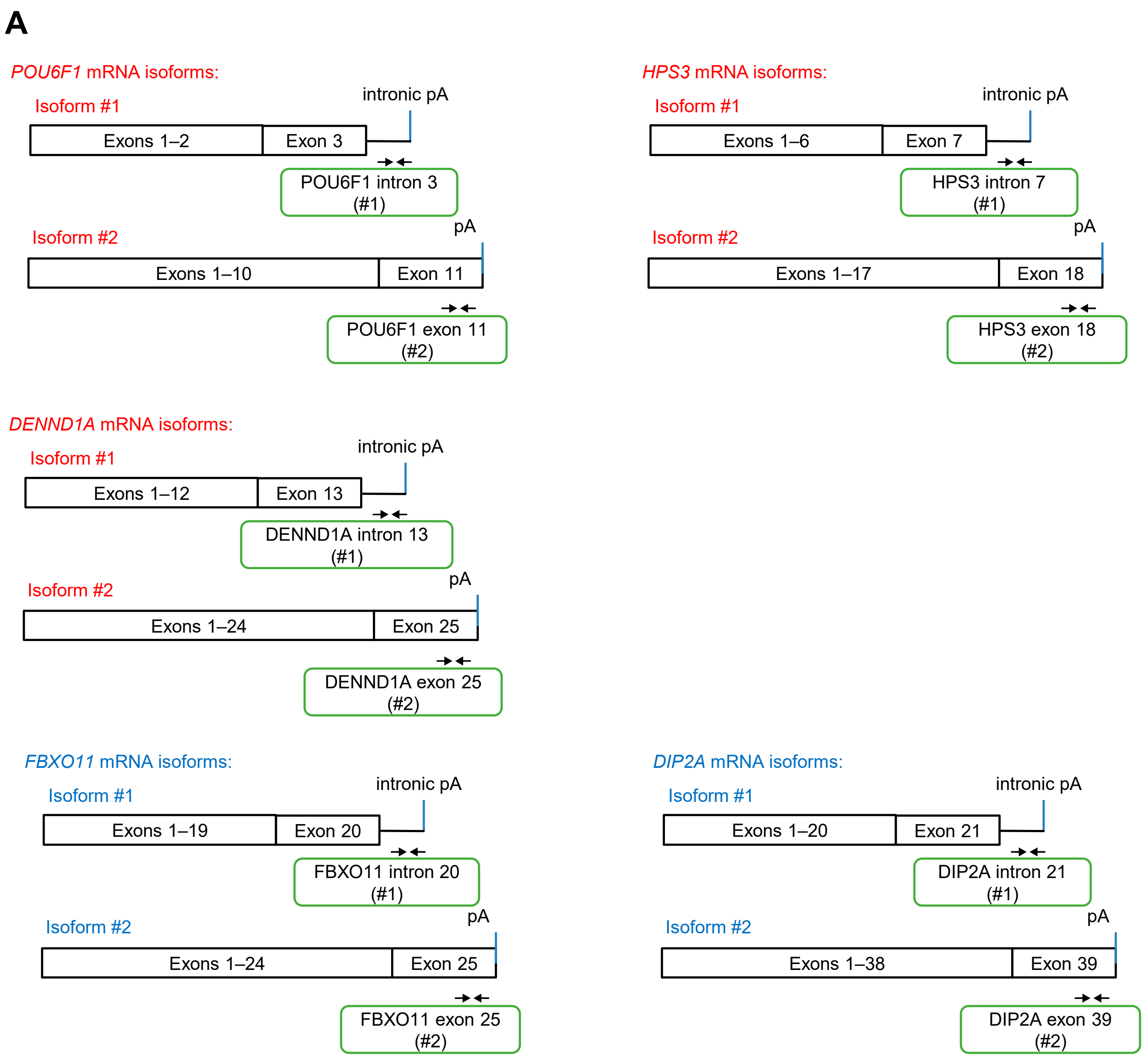
A
POU6F1 mRNA isoforms:
Isoform #1
intronic pA
Exons 1–2
Exon 3
POU6F1 intron 3 (#1)
Isoform #2
pA
Exons 1–10
Exon 11
POU6F1 exon 11 (#2)
HPS3 mRNA isoforms:
Isoform #1
intronic pA
Exons 1–6
Exon 7
HPS3 intron 7 (#1)
Isoform #2
pA
Exons 1–17
Exon 18
HPS3 exon 18 (#2)
DENND1A mRNA isoforms:
Isoform #1
intronic pA
Exons 1–12
Exon 13
DENND1A intron 13 (#1)
Isoform #2
pA
Exons 1–24
Exon 25
DENND1A exon 25 (#2)
FBXO11 mRNA isoforms:
Isoform #1
intronic pA
Exons 1–19
Exon 20
FBXO11 intron 20 (#1)
Isoform #2
pA
Exons 1–24
Exon 25
FBXO11 exon 25 (#2)
DIP2A mRNA isoforms:
Isoform #1
intronic pA
Exons 1–20
Exon 21
DIP2A intron 21 (#1)
Isoform #2
pA
Exons 1–38
Exon 39
DIP2A exon 39 (#2)

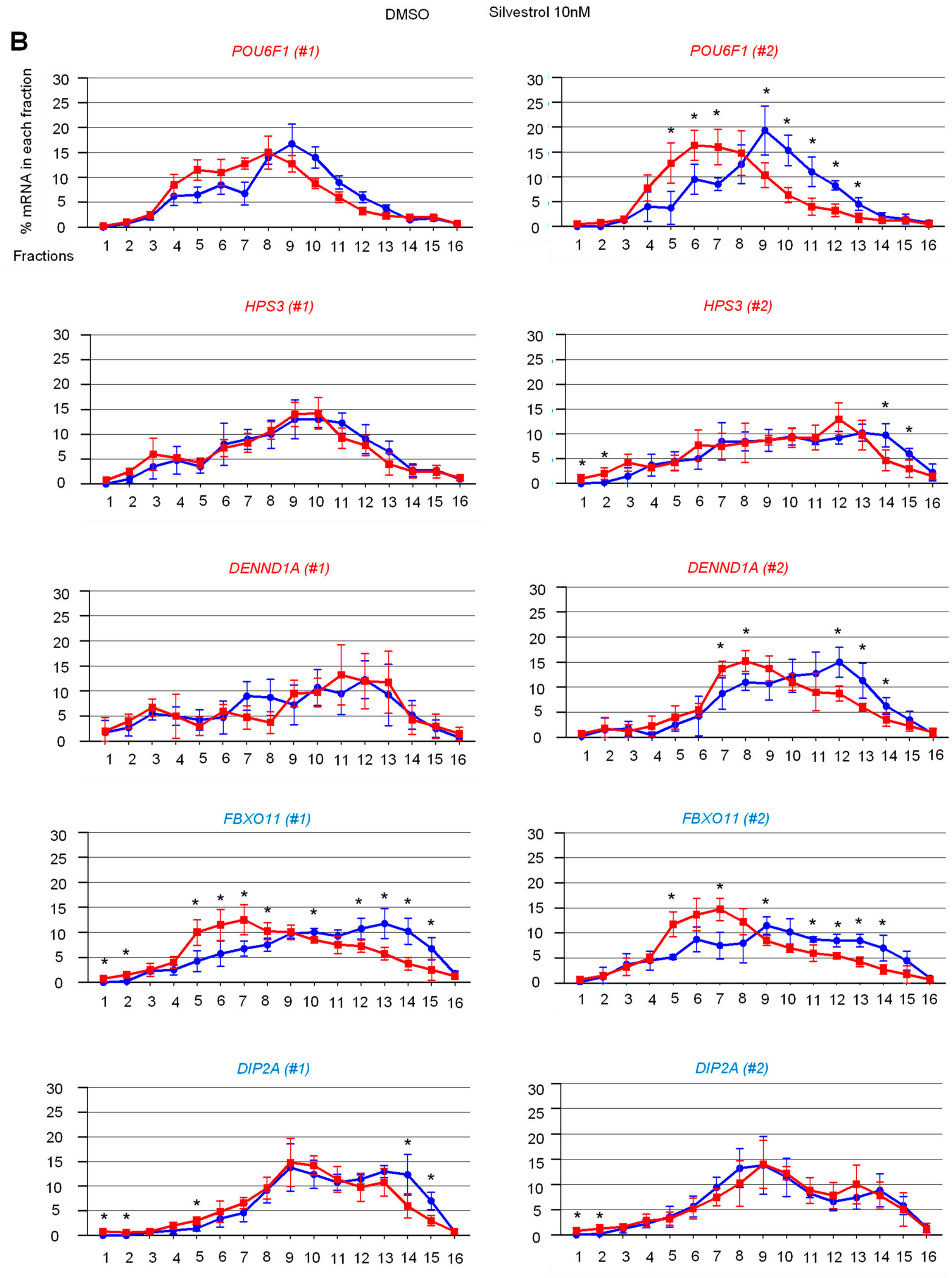

Figure S7. (A) Primer design for immune-related gene mRNA isoforms of IPA:LE UP (red) and IPA:LE DOWN (blue) candidates. (B) Percentage of transcripts for each APA isoform in each polysomal fraction obtained by sucrose-gradient ultracentrifugation was quantified by RT-qPCR in Jurkat cells ($n = 3$).

Figure 1C

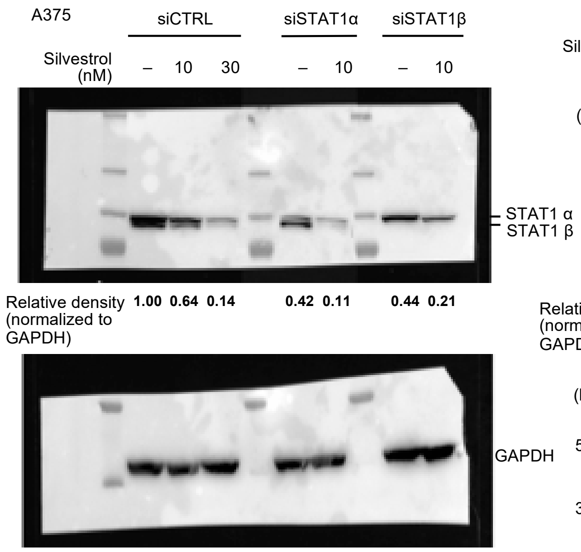

Figure 2D

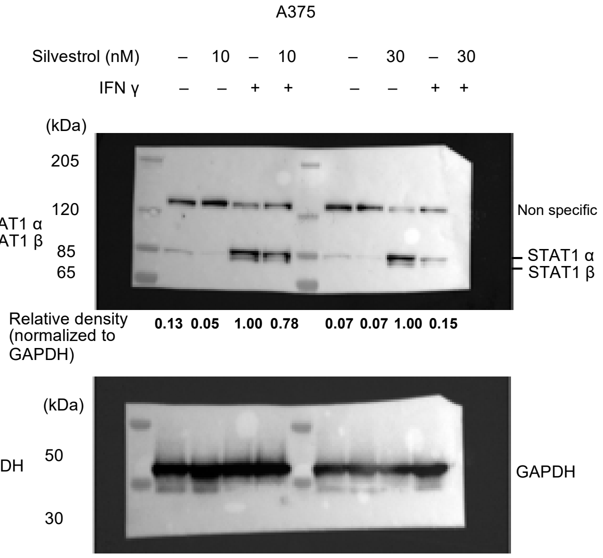

Figure 4E

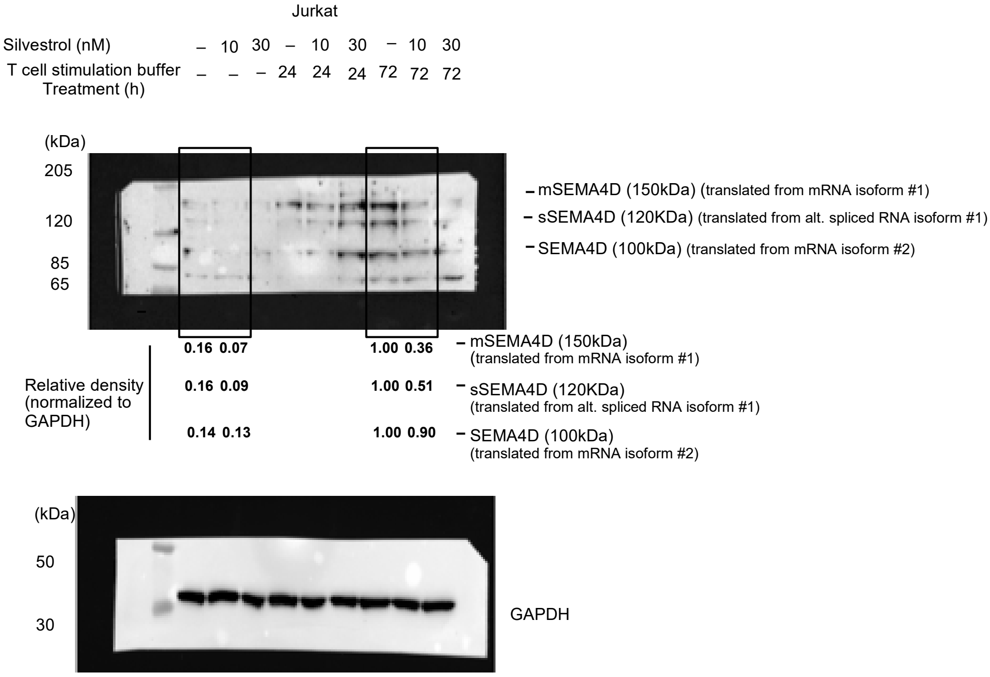

Figure S1C

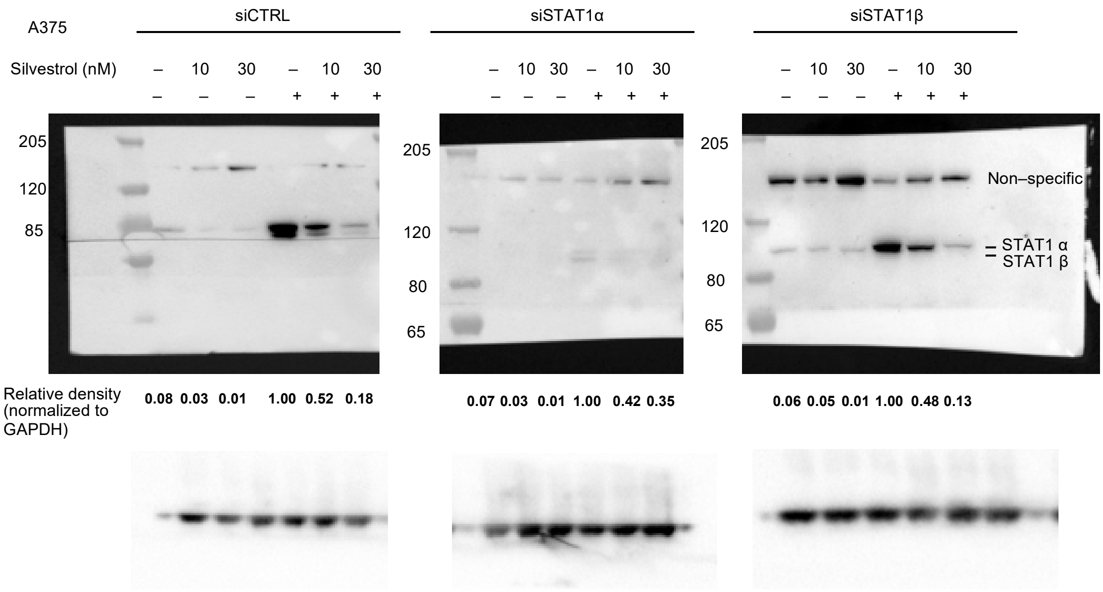

Figure S4

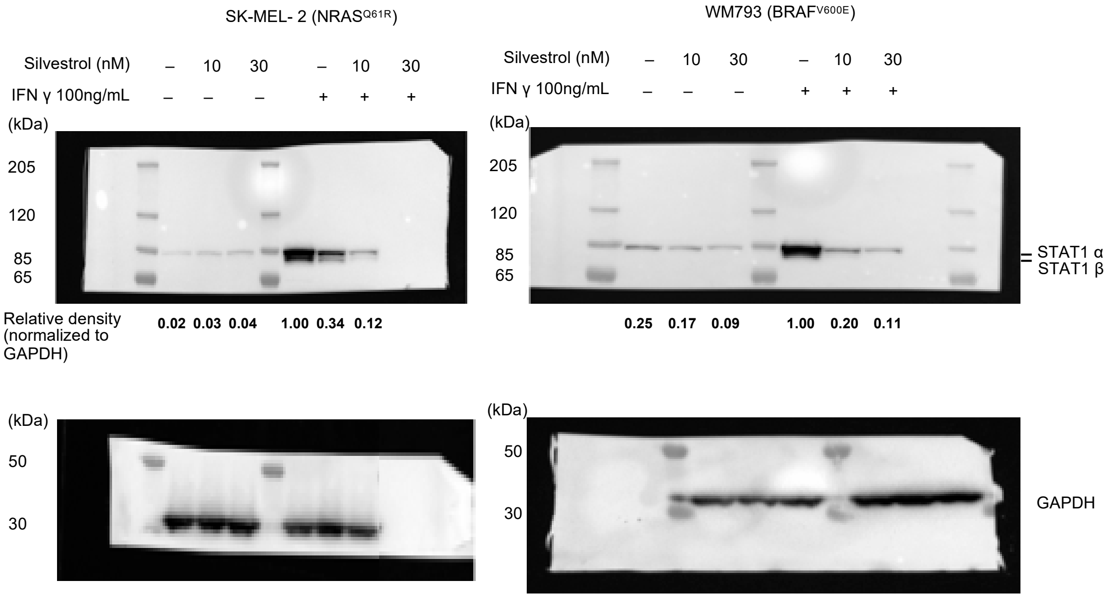

Figure S8. Uncropped Western Blot original images.

Table S1. Primer sequences used for RT-qPCR analysis of all mRNAs investigated in this paper.

Primer Sequences	Forward	Reverse
STAT1*a*	AGTCAGTGCCCAACTGTTATAGG	CCGTTCCTACGTCAAGCAGT
STAT1*b*	ACTGCCTATCAGCATTTTACTACT	TCAAAGGGTATCAGTTTTGGGA
STAT1 ex.2_4	GTGGCAGGATGTCTCAGTGG	CAGCTGTGACAGGAGGTCAT
PD-1 exon 5 (#1 + #2)	CTCTGTGGACTATGGGGAGC	AGAGCAGTGTCCATCCTCAG

PD-1 exon 5 (#2)	CCTGGGTGGGAAGGTACAG	CCTTGTCCCAGCCACTCAG
CTLA-4 exon 4 (#1 + #2)	GCCCTCTTACAACAGGGGTCT	GGCTGAAATTGCTTTTCACATTCT
CTLA-4 exon 4 (#2)	CTCCAGGAGACCCACAGGTA	CTGCTGCCTTCTTCTGTCCA
TIM-3 exon 7 (#1 + #2 + #3)	CTCTTTGGCCAACCTCCCTC	CATTGCAAAGCGACAACCCA
TIM-3 exon 7 (#2 + #3)	GGCAAAGGCCTTCAGCAATC	AGGGGGCAACAATAAGCAAGA
TIM-3 exon 7 (#3)	TGGCTTAGGATTGACTTGGTG	CCATGCTCATGGGTAGGAAG
LAG-3 intron 5 (#1)	AGACTAGGCAAACCCACCCT	GAAAAGTGGGGGCCGAGAT
LAG-3 exon 8 (#2)	CGCAGGCTCAGAGCAAGATA	GCCTCAGCTCCAGGTCAGA
CD40-L exon 5 (#1 + #2)	AAACCTTGCGGGCAACAATC	TGACAAACACCGAAGCACCT
CD40-L exon 5 (#2)	GCCTGCCCACTCCTCATTAC	TCTGGAAACAATGGAGACTGC
CD137 exon 9 (#1 + #2)	GTAACACGACATGCTCCACC	GGACAAAGGCAGAAGGTGTG
CD137 exon 9 (#2)	TGGAAGATATCACTCTGACGGA	AAGTCCTACCACACACAGGG
CD27 exon 6 (#1 + #2)	TGTCATTACAGCTGCCCCAG	CAGGCTCCGGTTTTCGGTAA
CD27 exon 6 (#2)	TCGAGACTGGCAGGGACG	GCCCGTCTTGTAGCATGTG
BTLA exon 5 (#1 + #2)	GGTTTGTGTTACCTGCATCAGT	GGTTGGTTAGTCAAATTGGGACA
BTLA exon 5 (#2)	TTCCCAAATGACTCTACTTCACT	TCTCATTGAGCACAATCACAAGC
IDO1 exon 9 (#1 + #2)	CATCCTGATTCCTGCAAGCC	TCAGTGCCTCCAGTTCCTTTG
IDO1 exon 9 (#2)	ACCCATTGTAACAGAGCCACAA	TTCCTACAAGAAATGCACAGGT
ADAR-1 intron 4 (#1)	AACTGAGATTGCGCCACTG	AGGCTGGTCTCGAATTCCTC
ADAR-1 exon 17 (#2)	CTCCAAGAGCAGAGTGAGGA	GTCACTGTTATCAAGGGACACA
AHNAK intron 5 (#1)	TGGAGTGGAAGGAGACCTCG	CACATCCACAGTGTAGGCCG
AHNAK exon 7 (#2)	GTAGAAGCGGCCAGGAAGAA	GAACAATGCTCCAAAGAACGGT
SEMA4D intron 18 (#1)	CCAAAAATCCAACCTGGCCC	AAGACCGTACTTGGGGCTCT
SEMA4D exon 23 (#2)	TCAACCCCAACAAGACCCTGC	TCACAGTTGTGGGGGACCAG
POU6F1 intron 3 (#1)	TCCAGTCTTTGTCACACATGC	ACGTGGTGGTGTCATACTACT
POU6F1 exon 11 (#2)	TGAACTGCGGAACCAGGAAG	CGTTTGCGTTTCTTGGAGGG
HPS3 intron 7 (#1)	TTTTGACTAAAGCAGAACCT-<br>GAAGC	GATGCTTACAGAAGCCTCTTGG
HPS3 exon 18 (#2)	CCCAGAAGATGGTACTGCAACA	AGTCAATGGTTTCTTTCGACTGC
DENND1A intron 13 (#1)	AATGTGCAGCTGGAGGGAAA	TCTCCACATGGATCCCTAGC
DENND1A exon 25 (#2)	CTCGAAGGCCCCAGAACC	GTGATGCTGCCCAGAGTAGG
FBXO11 intron 20 (#1)	GCAATGGCTGGAGTCTGGAT	CAGATGCCACCATCTCTTCCA
FBXO11 exon 25 (#2)	ACACTATATGACTCTGCTCCACC	TGGCAGGACTTTTTCTTTAGGGA
DIP2A intron 21 (#1)	TTCATCGTGGGCAAACTGGA	GCCACAACGTCATCTGCATT
DIP2A exon 39 (#2)	GAGCAGGATGCCCTGGAC	GTCCACGATGACCACCACTC
TBP	TGACCTAAAGACCATTGCACTTCG	CGTGGTTCGTGGCTCTCTTATC

HPRT	GCTGAGGATTTGGAAAGGGTGT	CCATCTCCTTCATCACATCTCG
